# Driving Cessation and Cognitive Dysfunction in Patients with Mild Cognitive Impairment

**DOI:** 10.3390/jcm7120545

**Published:** 2018-12-13

**Authors:** Jung-Min Pyun, Min Ju Kang, Sohee Kim, Min Jae Baek, Min Jeong Wang, SangYun Kim

**Affiliations:** Department of Neurology, Seoul National University College of Medicine and Seoul National University Bundang Hospital, Seongnam-si 13620, Korea; pyun.jungmin5@gmail.com (J.-M.P.); minju0321@naver.com (M.J.K.); ucb.sohee@gmail.com (S.K.); mjbaek1208@hanmail.net (M.J.B.); wangmj98@gmail.com (M.J.W.)

**Keywords:** driving cessation, mild cognitive impairment, cognition, working memory

## Abstract

Although driving by adults with cognitive impairment is an important public health concern, little is known about the indicators of driving cessation in patients with mild cognitive impairment (MCI). We aimed to investigate the prevalence of driving cessation in patients with MCI and the predictive value of cognitive performances for driving cessation. Patients with MCI were recruited in the Seoul National University Bundang Hospital; they met following inclusion criteria. Age range of 51–80 years, Clinical Dementia Rating scale score of 0.5, and ever car drivers including former and current drivers. All participants underwent comprehensive standardized cognitive assessments and information on driving status was obtained via an interview using a systematic questionnaire. The median age of the 135 participants was 72 years, and 54 participants (40%) were women; 93 patients (68.9%) were current drivers and 42 (31.1%) were former drivers. In univariate analysis, former drivers showed poorer performances in digit span backward and categorical fluency tests than current drivers. In multivariate logistic regression analysis, a poor digit span backward test score was significantly related with driving cessation (odds ratio: 0.493, 95% confidence interval: 0.258–0.939). In patients with MCI, poor performance in the digit span backward test, which represents impaired working memory capacity, was associated with a higher probability of driving cessation.

## 1. Introduction

The driving ability of patients with cognitive impairment is an important public health concern. Approximately 22–46% of patients with mild to moderate dementia drive [1,2]. Although driving allows the maintenance of mobility, independence, and functional daily living [3,4], patients with dementia are at risk of accidents due to their impaired cognition [5,6,7]. In this regard, it is critical to know the factors that predict driving cessation in patients with dementia. Previous studies have revealed that driving cessation in patients with dementia was related with older age, female sex, and lower cognitive and functional ability [2,8,9,10]. A longitudinal study suggested the rate of worsening of dementia and functional ability as the specific predictors of driving cessation in patients with dementia [11]. Patients with mild cognitive impairment (MCI) also have cognitive impairment but with preserved independence in their everyday functional abilities [12]. It can be considered that they present lesser risk during driving than patients with dementia. However, some patients with MCI stop driving due to cognitive problems, and several studies have demonstrated that compared with cognitively intact individuals, patients with MCI have impaired driving skills, such as poor lane and speed control [7,13,14].

Nevertheless, since daily functional ability is not compromised in patients with MCI, it is difficult to know when they should be cautious about driving or when they should stop driving altogether. This difficulty highlights the importance of investigating the driving status and the influential factors for driving cessation in patients with MCI. Despite great interest, little is known about the prevalence of driving cessation in patients with MCI and the characteristics of their cognitive performance.

Therefore, we aimed to (1) assess the prevalence of driving cessation in patients with MCI, (2) the difference in cognitive performance between current and former drivers, and (3) determine the specific cognitive domains that are potential predictors of driving cessation.

## 2. Materials and Methods

### 2.1. Study Subjects

A cross-sectional study was conducted in a memory clinic in the Seoul National University Bundang Hospital in the Republic of Korea. Between January 2015 and September 2016, participants who met the following inclusion criteria were recruited, (1) aged between 51 and 80 years, (2) a diagnosis of MCI according to the diagnostic criteria for MCI due to Alzheimer’s disease of the National Institute on Aging-Alzheimer’s Association [12] and a Clinical Dementia Rating scale (CDR) [15] score of 0.5, and (3) had ever driven a car including former and current drivers. Based on a systematic interview, participants were excluded if they had stopped driving but not due to cognitive impairment. All participants or their legal representatives provided written informed consent. The study was conducted in line with the principles of the Declaration of Helsinki and approved by the Institutional Review Board of Seoul National University Bundang Hospital (IRB Number B-1508/312-307).

### 2.2. Driving History

Former drivers completed a structured questionnaire about the reason for driving cessation. The answers were categorized into (1) cognitive impairment, such as deficits of attention, judgment, or ability to respond while driving; (2) financial problems; (3) physical problems; (4) the suggestion of family or friends; (5) traffic accident by the patient’s mistake; and (6) traffic accident by the opponent’s mistake. Participants could choose multiple answers and were required to explain the chosen answers in detail, as follows. What kind of physical problem in response to (3), and the reason for the suggestion to cease driving in response to (4). We excluded participants whose reasons for driving cessation were (2) financial problems, (3) physical problems, or (6) traffic accidents caused by an opponent’s mistake, or a combination of these.

### 2.3. Cognitive Assessment

At the same time as the interview about the driving history, extensive neuropsychological assessments were conducted, which included tests to measure attention, language, verbal and visual memory, visuoconstructive function, and frontal executive function. We used the following tests. The Digit Span Test for attention [16], the Korean version of Boston Naming Test for language [17], Seoul Verbal Learning Test for verbal memory [18], Rey Complex Figure Test (RCFT) for visuoconstructive function and visual memory [19], categorical fluency test of the Controlled Oral Word Association Test [20], and color reading of the Stroop test for executive function [21]. The subset scores were converted to standardized scores (Z-scores), which were adjusted for age, sex, and education level. Additionally, we used the Mini-Mental State Examination (MMSE) as a measure of global cognition [22], CDR Sum-of-Boxes (CDR-SB) to assess clinical severity [15], and the short form of the Geriatric Depression Scale (GDpS) [23].

### 2.4. Statistical Analysis

To compare the demographic data and neuropsychological test scores, we used the Pearson chi-squared test for categorical variables and the Mann–Whitney test for continuous variables. Binary logistic regression analysis was performed to identify the factor associated with driving cessation in three separate models. A total of 135 participants were included for analysis in the models. In Model 1, demographic characteristics (age, gender, and education level) and global cognitive status (the MMSE and CDR-SB score) were entered as independent variables and driving cessation was entered as the dependent variable. In Model 2, the scores of the neuropsychological assessments were entered as independent variables and driving cessation was entered as the dependent variable. In Model 3, the variables that were significant in Models 1 and 2 (age, gender, and digit span backward test score) were entered as independent variables and driving cessation was entered as the dependent variable. The variance inflation factors (VIFs) were calculated among the included variables to evaluate multicollinearity. The equations of the models were as follows:

Model 1: Logit (p) = b0 + b1 × age + b2 × gender (female) + b3 × education + b4 × MMSE + b5 × CDR-S

Model 2: Logit (p) = b0 + b1 × Digit span forward + b2 × Digit span backward + b3 × Korean Boston Naming Test + b4 × Seoul verbal learning test (Learning) + b5 × Seoul verbal learning test (Delayed recall) + b6 × Seoul verbal learning test (Recognition) + b7 × Rey complex figure test (Copy) + b8 × Rey complex figure test (Delayed recall) + b9 × Rey complex figure test (Recognition) + b10 × Categorical fluency + b11 × Stroop test colors

Model 3: Logit (p) = b0 + b1 × age + b2 × gender (female) + b3 × Digit span backward

Statistical significance was set at ≤0.05. We used IBM Statistical Package for the Social Sciences version 22 (IBM Corp., Armonk, NY, USA) for the analyses.

## 3. Results

### 3.1. Subject Characteristics and Reason for Driving Cessation

A total of 137 patients with MCI were recruited in the study. Among them, two patients were excluded from the analysis because they stopped driving due to poor physical condition (stroke and macular degeneration). The median age of the 135 participants was 72 years, and 54 participants (40%) were women. Among them, 93 participants (68.9%) were current drivers and 42 (31.1%) were former drivers. The causes of driving cessation among the former drivers are shown in Table 1. Cognitive impairment, such as deficits of attention, judgment, or the ability to respond when driving, was the most frequent reason. In the interview, we confirmed that the suggestion of family or friends to stop driving was based on the risky driving behavior of the patients.

### 3.2. Demographic Characteristics and Cognitive Assessment of Current and Former Drivers

The demographic and cognitive characteristics of the current and former drivers are summarized in Table 2. The former drivers were older and there were more women compared with the current drivers. Global cognitive functioning, which was indicated by MMSE score (28 vs. 27 in the current and former drivers, respectively, *p* = 0.041) and CDR-SB score (1.00 vs. 1.25 in the current and former drivers, respectively, *p* = 0.017) was poorer and the GDpS score (3 vs. 5 in the current and former drivers, respectively, *p* = 0.008) which represents depressive symptoms, was higher in the former drivers. From extensive neuropsychological tests, we found that former drivers showed poorer performance in the digit span backward test (−0.89 vs. −0.26, *p* = 0.029) and categorical fluency test (−0.92 vs. −0.48, *p* = 0.013) than the current drivers. A tendency to show declined ability in RCFT in terms of delayed recall was observed in the former drivers, but this was not statistically significant (−0.97 vs. −0.70, *p* = 0.088).

### 3.3. Predictors Associated with Driving Cessation

Table 3 shows the multivariate logistic regression analysis of the three models. In Model 1, older age (odds ratio (OR), 1.17, 95% confidence interval (CI), 1.07–1.29) and the female gender (OR, 13.01, 95% CI, 4.72–35.88) were significantly associated with driving cessation. In Model 2, the digit span backward test score was significantly associated with driving cessation (OR, 0.49, 95% CI, 0.25–0.93). The VIFs were less than 2.737 for all variables in Model 2, indicating a low degree of collinearity. In Model 3, age, female gender, and poor digit span backward test score were all found to increase the risk for driving cessation (OR, 1.17, 95% CI, 1.06–1.28, OR, 14.33, 95% CI, 5.05–40.66, and OR, 0.54, 95% CI, 0.31–0.95, respectively).

## 4. Discussion

The aims of this study were to investigate the prevalence of driving cessation in patients with MCI, the cognitive functioning of current and former drivers, and determine the specific cognitive domains that are potential predictors of driving cessation. Our results showed that the rate of driving cessation in patients with MCI was 31.1%. To our knowledge, this is the first study to investigate the prevalence of driving cessation in patients with MCI based on extensive neuropsychological tests. Previously, a study reported a 17.2% prevalence of driving cessation in patients with MCI [24]. This low value compared to our result could be due to different diagnostic criteria for MCI or different profiles of detailed cognitive domains of the studied population. Another study with cognitively impaired but not demented older adults reported that 13.1% of former drivers showed impairment in a single cognitive domain and 36.5% showed impairment in multiple cognitive domains [25]. In addition, Vaughan et al. reported that the rate of former drivers among older women with MCI was 32.5% [26]. However, studies of the prevalence of driving in patients with MCI are scarce, and this issue deserves more attention since patients with MCI may lack awareness of their possibly risky driving behavior.

Our Model 1 multivariate analysis showed that older age and female gender were associated with driving cessation, which is concordant with previous reports. This gender effect has been explained by women being more engaged in self-regulating their driving [27,28], by women driving less frequently [29], and by the perception of driving being related more with masculinity [30].

In the Model 2 multivariate analysis, a poor score in the digit span backward test was associated with an increased risk of driving cessation, indicating that attention is a potential predictor of driving cessation in patients with MCI. In the Model 3, poor digit span backward test performance remained a significant risk factor for driving cessation independent of age and female gender.

The neuropsychological characteristics of cognitively impaired adults who have ceased driving have been studied mostly in patients with dementia. Although various cognitive domains are involved in driving ability, visuospatial function and attention are particularly important for driving performance [31,32,33,34,35]. In contrast, there has been little research into the cognitive functioning of patients with MCI regarding driving. According to a previous study, older women aged 65–79 years having MCI with functional limitations in instrumental activities of daily living, such as household tasks or grasping situation, were less likely to continue driving [26]. In a simulation study, poor driving skill in patients with MCI during a car-following task was correlated with poor Trail Making Test Part B score that represents visual attention and executive function [36].

In our study, it is of note that the digit span backward test score was predictive of driving cessation in patients with MCI. The digit span backward test is considered a useful tool to assess attention and working memory [16]. Working memory is the ability to maintain and manipulate information over a limited time [37]. Engle et al. stated that working memory is not defined by the storage or memory but by the capacity for controlled and sustained attention in the face of distraction [38]. This working memory capacity is important for maintaining goal-directed behavior in the presence of interference, such as while driving [39]. Among the various brain regions activated during working memory, the frontoparietal neural network is a common pathway involved in working memory that is independent of the type of information [40].

Driving is a cognitively challenging activity that requires fast and precise processing of new information and decision-making. Adapting to dynamically changing situations in the road traffic environment and integrating the new information demands a certain level of working memory capacity [41]. Several studies have shown the relation between working memory and driving. Increased working memory load while driving leads to impairment in lane changing performance [42] and increased response latency in braking reactions [43]. Functional studies have demonstrated that increased working memory load during driving is related to neural activity in the inferior frontal area [44,45]. The necessity of working memory capacity for safe driving is in line with our study’s finding that there was a significant association between the poor digit span backward test score and driving cessation. Furthermore, the predictive value of the digit span backward test score could be clinically applicable during consultation with patients having MCI with an aim to assess their driving performance. In a study with cognitively impaired but not demented older adults, over 60% of current drivers had restricted their driving, and ~98% of current drivers considered to restrict or quit driving in the future under certain circumstances, with the most popular reasons being doctor’s advice and danger to others [25]. This suggests that consultation about driving cessation based on objective assessment is important in patients with MCI.

Some limitations of this study are that the sample size was small and that the study was conducted in a single center. In addition, the participants were patients who visited the tertiary medical center, and they may differ from patients with MCI in a community-based population. Further, our result was not corrected for multiple comparisons. When multiple hypotheses are tested on the same issue, individual *p*-values of the tests may not be ideal indicators of actual statistical significance [46]. However, adjustment for multiple tests increases the likelihood of type II errors and the interpretation of the finding depends on the number of tests performed. Thus, merely describing the tests of significance that were performed is also a way of dealing with multiple comparisons in an exploratory study [47]. Moreover, even though we confirmed in the interview about driving history that the driving cessation is due to cognitive decline, there is the possibility that cognitive decline progressed after the driving cessation. In a further longitudinal study, we could overcome this by evaluating the association between the incidence of driving cessation and the change in cognitive function at baseline and the follow-up.

In future studies, it would be meaningful to investigate whether our results correlate with the results of an on-road assessment. A recent meta-analysis suggested that patients with a CDR score of 0.5 showed an 11–12% failure rate in the on-road assessment [48]. Understanding the correlation between attention ability and the on-road assessment could provide supporting evidence for driver assessment in patients with MCI.

## 5. Conclusions

In summary, our study demonstrated that the prevalence of driving cessation in patients with MCI is 31.1% and that driving cessation is associated with impairment in the digit span backward test, which reflects poor attention and working memory. Despite the potential limitations, this study supports the importance of working memory, among other cognitive domains, for safe driving and the findings will help clinicians counsel patients with MCI about their driving ability.

## Figures and Tables

**Table 1:** Reasons for driving cessation.

Reasons	Number of Patients
1. Cognitive impairment, such as deficits in attention, judgment, or the ability to respond when driving	34
2. Financial problems	0
3. Physical problems	2
4. Suggestion of family or friends	3
5. Accident was due to the patient’s mistake	6
6. Accident was due to the opponent’s mistake	0

**Table 2:** Demographic characteristics and cognitive assessments of current and former drivers with mild cognitive impairment (MCI).

Variable	Current Driver (*n* = 93)	Former Driver (*n* = 42)	*p*-Value
Age	71.00 (65.00–75.00)	73.00 (69.00–76.00)	0.017
Female, n (%)	25 (26.88)	29 (69.05)	<0.001
Education, years	13.50 (10.00–16.00)	16.00 (12.00–16.00)	0.115
Amnestic MCI, n (%)	46 (49.46)	22 (52.38)	0.898
Cognitive Assessment			
MMSE (range 0–30)	28 (26–29)	27 (26–28)	0.041
CDR-SB (range 0–9)	1.0 (0.5–4.5)	1.25 (0.5–2.0)	0.017
GDpS (range 0–15)	3 (0.5–5.5)	5 (2–7.5)	0.008
Digit Span Forward Test	0.03 (−0.79–0.74)	−0.17 (−0.97–0.58)	0.431
Digit Span Backward Test	−0.26 (−0.91–0.42)	−0.89 (−1.18–−0.04)	0.029
Korean Boston Naming Test	−0.44 (−1.09–0.37)	−0.81 (−1.35–0.12)	0.139
SVLT Learning	−0.89 (−1.28–−0.28)	−0.76 (−1.06–−0.42)	0.285
SVLT Delayed recall	−0.99 (−1.37–−0.12)	−1.13 (−1.41–−0.6)	0.510
SVLT Recognition	−0.63 (−1.22–0.13)	−0.61 (−1.32–0.32)	0.896
RCFT Copy	−0.57 (−1.18–0.16)	−0.29(−1.28–0.45)	0.713
RCFT Delayed recall	−0.70 (−1.24–0.03)	−0.97 (−1.37–−0.36)	0.088
RCFT Recognition	−0.64 (−1.22–0.1)	−0.25 (−0.74–0.16)	0.206
Categorical fluency	−0.48 (−1.09–0.41)	−0.92 (−1.28–−0.17)	0.013
Stroop Colors Test	−0.40 (−1.17–0.7)	−0.78 (−1.36–0.4)	0.234

Data are presented as the median (interquartile range) unless otherwise specified. MCI, mild cognitive impairment; MMSE, Mini-Mental State Examination; CDR-SB, Clinical Dementia Rating Sum of Boxes; GDpS, Geriatric Depression Scale score; SVLT, Seoul Verbal Learning Test; RCFT, Rey Complex Figure Test.

**Table 3:** Binary Logistic Regression Analysis to assess driving cessation-associated predictors.

Variable	Odds Ratio	95% Confidence Interval	*p*-Value
Model 1			
Age	1.17	1.07–1.29	0.001
Female	13.01	4.72–35.88	<0.001
Education	0.92	0.80–1.06	0.295
MMSE	1.09	0.82–1.45	0.531
CDR-SB	1.69	0.91–3.13	0.092
Model 2			
Digit Span Forward Test	1.17	0.65–2.12	0.583
Digit Span Backward Test	0.49	0.25–0.93	0.032
Korean Boston Naming Test	0.86	0.51–1.44	0.568
SVLT Learning	2.05	0.89–4.71	0.089
SVLT Delayed Recall	0.56	0.22–1.40	0.218
SVLT Recognition	1.33	0.69–2.54	0.386
RCFT Copy	1.60	0.92–2.80	0.095
RCFT Delayed Recall	0.49	0.24–1.00	0.052
RCFT Recognition	1.68	0.92–3.06	0.088
Categorical Fluency	0.57	0.30–1.07	0.083
Stroop Colors Test	0.91	0.55–1.53	0.742
Model 3			
Age	1.17	1.06–1.28	0.001
Female	14.33	5.05–40.66	<0.001
Digit Span Backward Test	0.54	0.31–0.95	0.032

MMSE, Mini-Mental State Examination; CDR-SB, Clinical Dementia Rating Sum of Boxes; SVLT, Seoul Verbal Learning Test; RCFT, Rey Complex Figure Test.
